# Cancer Cell Analyses at the Single Cell-Level Using Electroactive Microwell Array Device

**DOI:** 10.1371/journal.pone.0139980

**Published:** 2015-11-11

**Authors:** Marina Kobayashi, Soo Hyeon Kim, Hiroko Nakamura, Shohei Kaneda, Teruo Fujii

**Affiliations:** 1 Institute of Industrial Science, The University of Tokyo, Tokyo, Japan; 2 LIMMS/CNRS-IIS UMI 2820, The University of Tokyo, Tokyo, Japan; 3 JST CREST, Tokyo, Japan; Texas A&M University, UNITED STATES

## Abstract

Circulating tumor cells (CTCs), shed from primary tumors and disseminated into peripheral blood, are playing a major role in metastasis. Even after isolation of CTCs from blood, the target cells are mixed with a population of other cell types. Here, we propose a new method for analyses of cell mixture at the single-cell level using a microfluidic device that contains arrayed electroactive microwells. Dielectrophoretic (DEP) force, induced by the electrodes patterned on the bottom surface of the microwells, allows efficient trapping and stable positioning of single cells for high-throughput biochemical analyses. We demonstrated that various on-chip analyses including immunostaining, viability/apoptosis assay and fluorescent in situ hybridization (FISH) at the single-cell level could be conducted just by applying specific reagents for each assay. Our simple method should greatly help discrimination and analysis of rare cancer cells among a population of blood cells.

## Introduction

Circulating tumor cells (CTCs), shed from primary and metastatic tumors and flowing into the blood, are considered as a major cause of cancer metastasis [1]. Counting the number of CTCs in peripheral blood makes it possible to monitor therapeutic effect and prognosis [2]. A challenge in detection of CTCs in a blood sample is that the existence of CTCs is extremely rare and mixed with normal blood components (1 in 10^9^ blood cells). Microfluidic devices are suitable for sorting and analysis of rare cells since one can efficiently handle complex cellular fluids with minimal damage to suspended cells [3, 4]. In addition, the ability of microfluidic devices to deal with the large volume of whole blood samples has already been shown [5]. Recently, several groups have been developing microfluidic devices to isolate CTCs from normal blood components, for example, by using antibody coated microposts, dielectrophoresis, size-based separation by a microfilter or acoustophoresis, etc. [6–11]. Although previous methods using microfluidic devices successfully demonstrated separation of CTCs, the separated cells have to be collected and preferably be analyzed at the single-cell level.

A practical issue on the CTC analysis is that the cancer cells are mixed with normal blood cells even after isolation of CTCs from blood. The previous CTC isolation methods show trade-off between recovery of CTCs and depletion of white blood cells (WBCs) [9–11]; the higher recovery rate of CTCs, the lower depletion rate of WBCs. These results indicate that the isolated cancer cells are still mixed with large number of WBCs. For instance, a microfluidic method using magnetophoretic WBC depletion allows 3.8-log depletion of WBCs and a 97% yield of cancer cells [12]. If an original blood sample contains 10-cancer cells and 10^6^-WBCs, a purified sample contains 10-cancer cells and 156-WBCs after isolation with the magnetophoretic WBC depletion method. Hence, after isolation of target cells from blood, discrimination between cancer cells and WBCs is highly required to detect or analyze the target cells.

Immunostaning or fluorescent in situ hybridization (FISH) is widely used method for the discrimination of cancer cells. However, conventional protocols using a test tube or a microliter plate require large volume of reagents, including antibodies or probes for the hybridization. Moreover, centrifugations, required for changing reagents of each assay, possibly cause critical loss of original samples, or damage on cell viability as well as cell function because of strong centrifugal forces acting on a cell [13–15]. A simple and efficient method for biochemical assay is therefore highly desirable to decrease possible risks of the conventional methods.

Here, we propose a new method for on-chip single-cancer cell analyses using electroactive microwell array (EMA) device. The EMA contains patterned thin-film electrodes on the bottom of each microwell for single-cell trapping with dielectrophoresis (DEP) [16, 17]. Since DEP force provides fast, active and stable trapping, we could efficiently trap cancer cells suspended in sample solution. Trapped cells can be stably held on a chip by DEP, allowing rapid exchange of reagents with an extremely small sample volume. Thus, high-throughput biochemical assays for arrayed single cells are facilitated. We demonstrated the feasibility of our approaches with a mixture of different cell types by carrying out three kinds of assays; cancer cell discrimination by immunostaining, viability/apoptosis assay and fluorescent in situ hybridization (FISH) analysis. The whole process for assays requires just sequential injection of cell suspension and reagents for the analyses without complicated valve or tubing systems. We expect our simple method facilitates high-throughput and parallel single cell analyses, while eliminating extra cell manipulations outside the device.

## Electroactive Microwell Array

### Design

**Fig 1:**
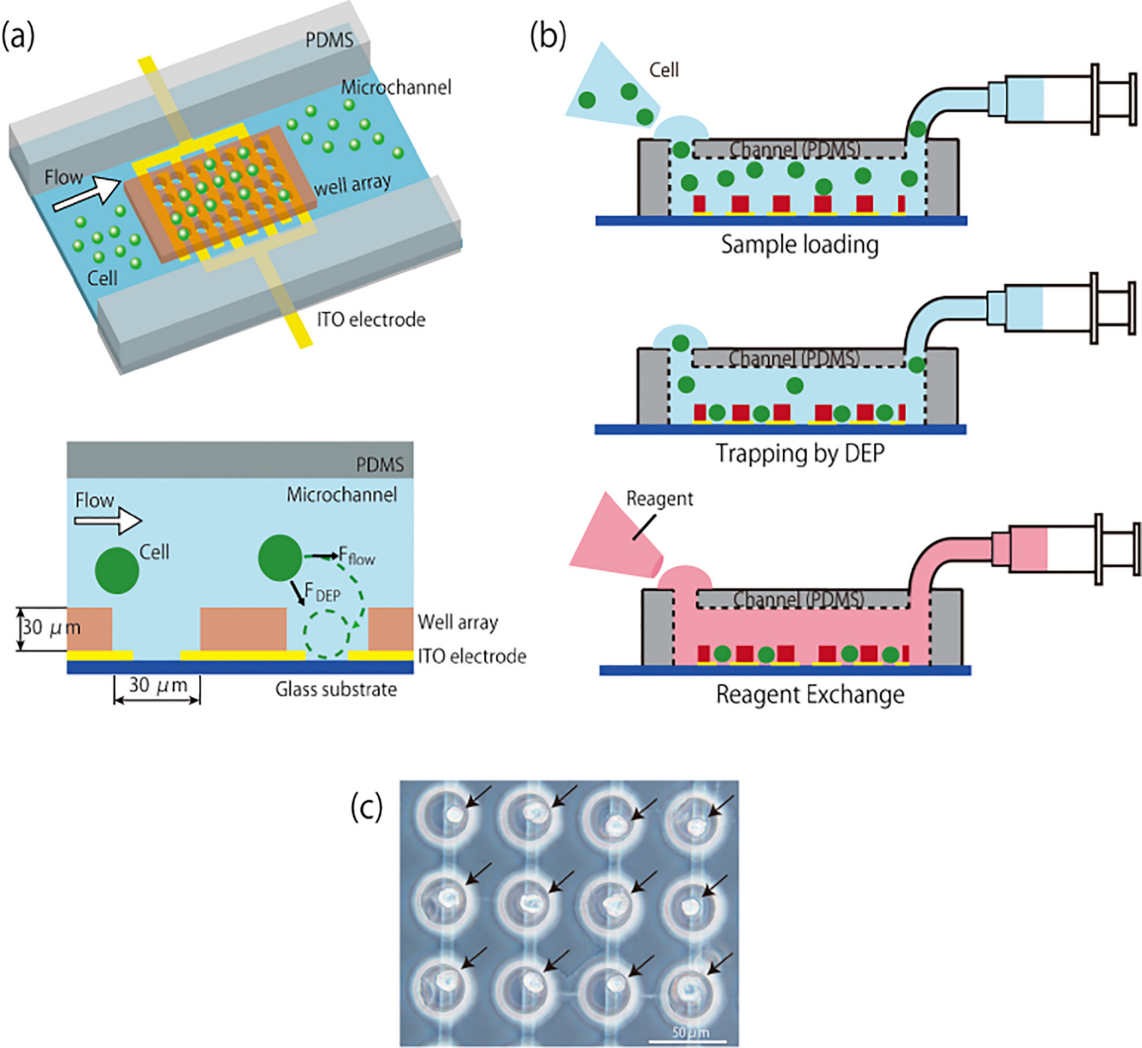
Concept of single cancer cell analysis using electroactive microwell array device. (a) Configuration of the device and dimensions of microwell and interdigitated electrodes. (b) Cell trapping and reagent exchange for single-cell analysis. Cell suspension is introduced into the microfluidic channel on the device and cells are trapped into the microwells by positive DEP. After cell trapping, analyses of the trapped cells are performed by introducing required reagents into the microchannel. (c) Image of trapped DU145 cells (indicated by arrows) in microwells.

The device consists of a microfluidic channel made of polydimethylsiloxane (PDMS), and a glass substrate that contains a large number of microwells fabricated on interdigitated indium tin oxide (ITO) electrodes (Fig 1A). The distance between the electrodes is about 6 μm and the diameter of microwells is 30 μm, which is bigger than the diameter of the target cells (20 μm). And the height of the microwell structure, which was made of epoxy resin, is 25 μm. The microwells are aligned with the interdigitated ITO electrodes in order to locate a pair of electrodes (anode and cathode) in each of the wells. One device contains 3168 microwells. Applied electric field is highly localized inside each microwell since the interdigitated electrodes are located at the bottom of the microwells. Fig 1B shows the procedure of single cell analysis. First, cells are introduced into the microchannel and trapped into the microwells using positive DEP induced by alternating voltage applied to the electrodes. Then, reagents for the analyses are introduced into the fluidic channel. Fig 1C shows the image of trapped cells in the microwells.

### Fabrication

**Fig 2:**
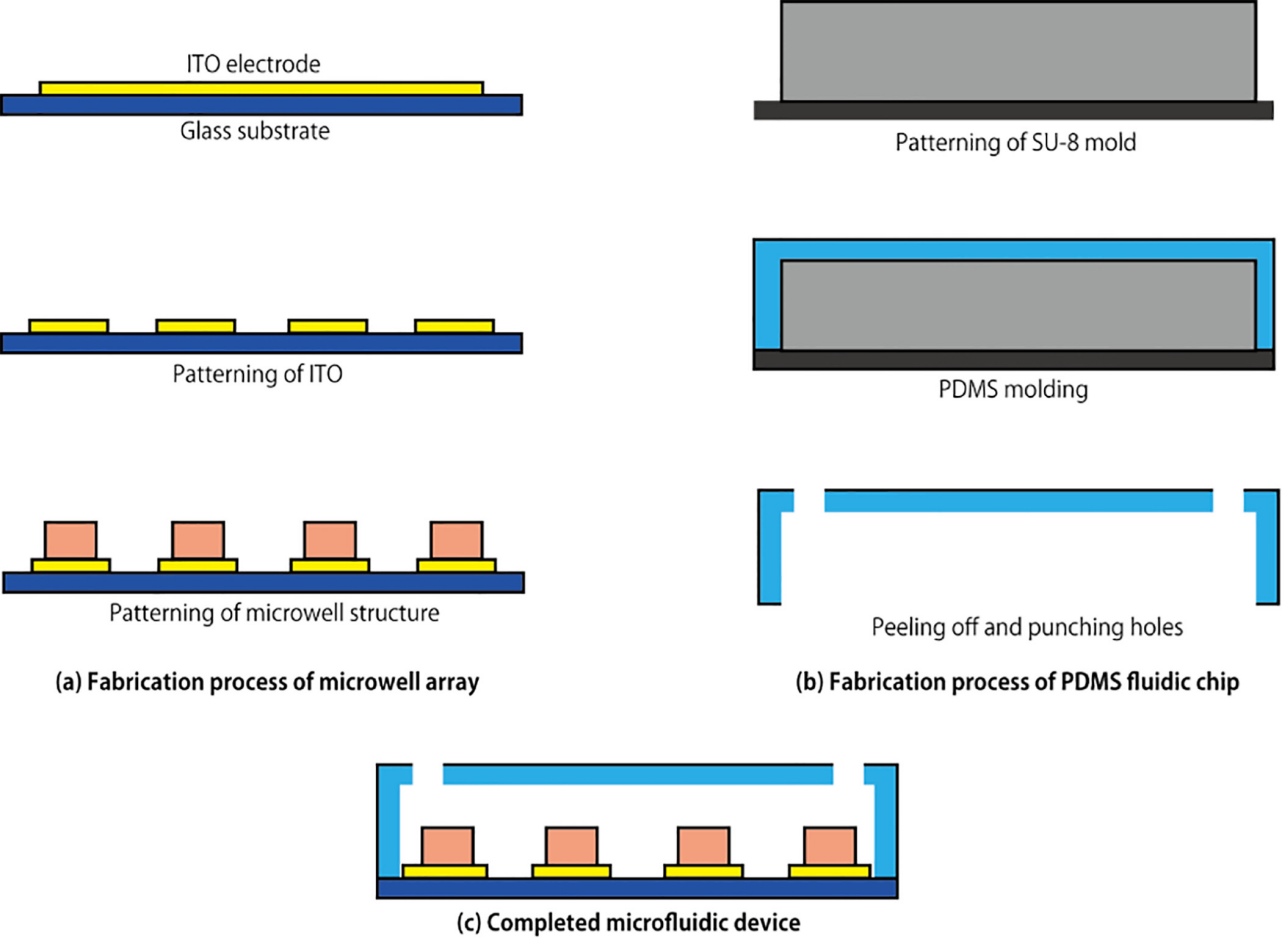
Fabrication process of the electroactive microwell array device. (a) Microwell array. The interdigitated electrodes are fabricated by a conventional patterning process of ITO and microwells made of KMPR are aligned with the electrode. (b) PDMS fluidic chip. The fluidic chip is fabricated through soft-lithography process. (c) Completed microfluidic device made by bonding two parts together.

Fig 2 shows the fabrication process of the present device. The shape of the electrodes were patterned using photoresist (AZP1350, AZ Electronic Materials) on a ITO coated glass substrate (TOA OPTICAL TECHNOLOGIES, LTD.), followed by etching of ITO by 0.2 M FeCl_3_ + 6 M HCl solution for 30 min at room temperature. After that, the substrate was cleaned and rinsed to remove the AZP1350 photoresist remaining on the ITO. The microwell array is fabricated with photoresist (KMPR1005, NIPPON KAYAKU CO.) on top of the patterned electrodes. The photoresist was spin-coated on the electrodes, and a chromium photo-mask patterned for the microwell array was aligned with the patterned ITO electrodes. The photoresist was exposed to ultraviolet light through the photo-mask, followed by development and rinsing.

The PDMS fluidic chip is fabricated through the standard replica molding process as shown in Fig 2B. Photoresist (SU-8 2100, MicroChem Co.), which serves as a mold, was patterned on a silicon wafer. The mold was thoroughly cleaned with isopropanol and deionized water. PDMS (Silpot 184, Dow Corning Toray, CO. Ltd.) was mixed with curing agent (10:1 mass ratio) and poured over the mold. Then, the PDMS was heated at 75°C for 1 hour followed by peeling off the polymerized PDMS from the mold. Holes as access ports to the flow channel were punched out.

In order to bond the microwell array and PDMS fluidic chip, they were exposed to O_2_ plasma to activate opposing surfaces using reactive ion etching machine (RIE-10NR, Samco CO.). Also O_2_ plasma treatment makes the KMPR microwells and the PDMS channel hydrophilic, which ensures easy injection of aqueous reagents into the channel and the microwells.

### Cell Trapping Using DEP Force

**Fig 3:**
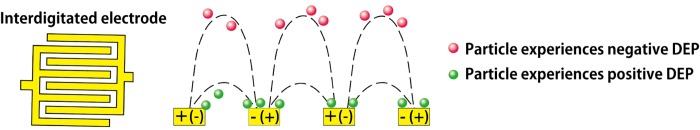
Movement of dielectric particles due to DEP force. Particles are attracted to the electrodes due to the positive DEP force, and repelled from the electrodes due to the negative DEP force.

In this study, cells introduced into the microchannel are actively trapped into microwell equipped with interdigitated electrode by DEP force. DEP is a phenomenon in which neutral particles move when it is applied to non-uniform electric field. The time-averaged DEP force can be approximated in terms of dipole effects as

$$F_{DEP}\;=\;2\pi \varepsilon_{m}r^{3}\text{Re}(f_{\text{CM}})\nabla {\text{E}}^{2},$$ (1)

where *ε*_*m*_ is absolute permittivity of the medium, *r* is radius of the particle, Re(*f*_CM_) is the real part of Clausius-Mossotti factor (*f*_CM_) relating to the induced dipole moment and E is the RMS value of the applied electric field. The *f*_CM_ is

$$f_{\text{CM}}=\;\frac{\varepsilon_{p}^{*}-\varepsilon_{m}^{*}}{\varepsilon_{p}^{*}+2\varepsilon_{m}^{*}},$$ (2)

$$\varepsilon_{m}^{*}=\;\varepsilon_{m}-i\frac{\sigma }{2\pi f}$$ (3)

$$\varepsilon_{p}^{*}=\;\varepsilon_{p}-i\frac{\sigma }{2\pi f}$$ (4)

where *ε*_*p*_ is absolute permittivity of the medium. The particle moves to a field maximum (positive DEP) or a field minimum (negative DEP) depending on the Re(*f*_CM_) which represents difference between the dielectric properties of the particle and its suspending medium (Fig 3) [18–20]. Re(*f*_CM_) can be controlled by adjusting the conductivity of the suspending medium and the frequency of applied electric field. Thus cells are trapped into microwell by positive DEP in the case of the EMA device [16].

## Materials and Methods

### Sample Preparation

U937 cells (leukemic monocyte lymphoma cell line, obtained from the RIKEN Bio Resource Center, Japan), DU145 cells (prostate cancer cell line, obtained from the RIKEN Bio Resource Center, Japan) and PC3 cells (prostate cancer cell line, obtained from the RIKEN Bio Resource Center, Japan) were cultured in a humidified incubator (37°C in an atmosphere of 5% CO_2_). The culture medium for all cells was RPMI 1640 (Invitrogen Corp.) supplemented with Fetal Bovine Serum (10%, Gemini Bio-products) and penicillin-streptomycin solution (1%, Sigma Chemical Co). The average diameter of U937 cell, DU145 cell and PC3 cell was 10 μm, 20 μm and 22 μm respectively. The cells were dispersed in a DEP buffer (10 mM HEPES, 0.01 mM CaCl_2_, 59 mM D-glucose and 236 mM sucrose; pH7.35) to adjust the conductivity of the cell suspension medium (21.4 mSm^-1^) for positive DEP [21]. The DEP buffer contained bovine serum albumin (1% wt/vol) to block nonspecific cell adhesion. Cells in the culture medium were centrifuged at 2000 rpm for 5 min. We gently removed the culture medium and added DEP buffer.

### Experimental Setup

The microfluidic device was mounted on the x-y translational stage located on inverted microscope (IX71, OLYMPUS). Cells were monitored with a camera (DP73, OLYMPUS), which was installed on the microscope. The electric potential for DEP was applied to the interdigitated ITO electrodes with the function generator (WF1974; NF Corp.) through an amplifier (HSA4101; NF Corp.).

### Immunostaining

Trapped cells in microwells were fixed with 4% paraformaldehyde in PBS (phosphate-buffered saline) for 10 minutes and washed with PBS for 5 minutes. Subsequently, they were permeabilized with 0.2% Triton X-100 in PBS for 5 minutes and washed with PBS for 5 minutes. Cells were immunostained with Hoechst33342 (DOJINDO) for DNA content, FITC-conjugated anti-cytokeratin antibodies (BD) for epithelial cells and PE conjugated anti-CD45 antibodies (Life technology) for leukocyte cells for 30 minutes. Finally the cells were rinsed with PBS for 10 minutes. The whole processes were conducted at a flow rate of 3 μL min^-1^.

### Viability and Apoptosis Assay

Trapped cells were exposed to Annexin V Alexa Fluor 488 (Invitrogen) at room temperature in the dark room for 30 minutes followed by exchanging the reagents into mixed reagents of Annexin binding buffer (Invitrogen), Propidium Iodide (Invitrogen) and calcein blue (Invitrogen) for 10 minutes. The whole process was conducted at a flow rate of 3 μL min^-1^.

### Fluorescent In Situ Hybridization (FISH)

Interphase FISH was performed on the trapped cells in the microwells according to the standard protocol with the following modifications. Briefly, trapped cells were fixed with Carnoy’s solution. The device was washed with 2×Saline-sodium citrate buffer (SSC, Abbott), dehydrated in an ascending series of alcohol, and air-dried. The probe mix (5’BCL-6 probe labeled in red and 3’BCL-6 probe labeled in green) for hybridization was added. DNA was denatured at 73°C for 5 min and then hybridized at 37°C for 16 hours on the thermal cycler (BECKMAN COULTER). After incubation with 0.4×SSC/ 0.3% Nonidet P-40 (NP-40) at 73°C, the device was transferred to 2×SSC/ 0.1% NP-40 at room temperature, it was air-dried in the dark room. DNAs were counterstained with DAPI (DOJINDO), sealed with cover glass.

## Results and Discussion

### Feasibility of the Device for On-Chip Immunostaining

**Fig 4:**
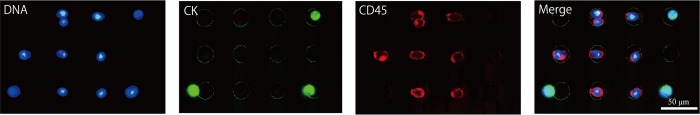
Cancer cell identification by immunostaining. Trapped DU145 cells and U937 cells stained with Hoechst33342 (blue), Anti-cytokeratin antibody (green) and Anti-CD45 antibody (red). Merged image identifies DU145 cells as a model of CTC (blue + green).

The feasibility of the present device for on-chip single-cancer cell analysis was demonstrated by carrying out immunostaining after trapping a mixture of two different cell lines including U937 cells (a model of white blood cell) and DU145 cells (a cancer cell line). The cell suspension was introduced into the device with a flow rate of 3 μL min^-1^. For the positive DEP, 10 Vp-p and sinusoidal electric potential at 8 MHz, was applied to the interdigitated ITO electrodes. After cell trapping with DEP, we carried out immunostaining to identify the trapped cells. Trapped cells were fixed, permeabilized and stained by sequential injection of the reagents into the device trough the access port. The cells were stained with Hoechst33342 (blue) staining DNAs, FITC-conjugated anti-cytokeratin antibodies (green) for epithelial cells and R-PE conjugated anti-CD45 antibodies (red) for leukocyte cells (Fig 4). Cytokeratin-positive cells were considered as a cancer cell, whereas CD45-positive cells were considered as WBC. Fig 4 shows trapped 11 cells (in 10 wells including 1 well with 2 cells trapped in upper left), 3 cells stained with anti-CK antibody (green) corresponding to cancer cell and 8 cells stained with anti-CD45 antibody (red) corresponding to WBC. In this way, identification of cancer cell and WBC can be done only by sequential injection of required reagents into the device without complex valve system or additional equipment.

**Fig 5:**
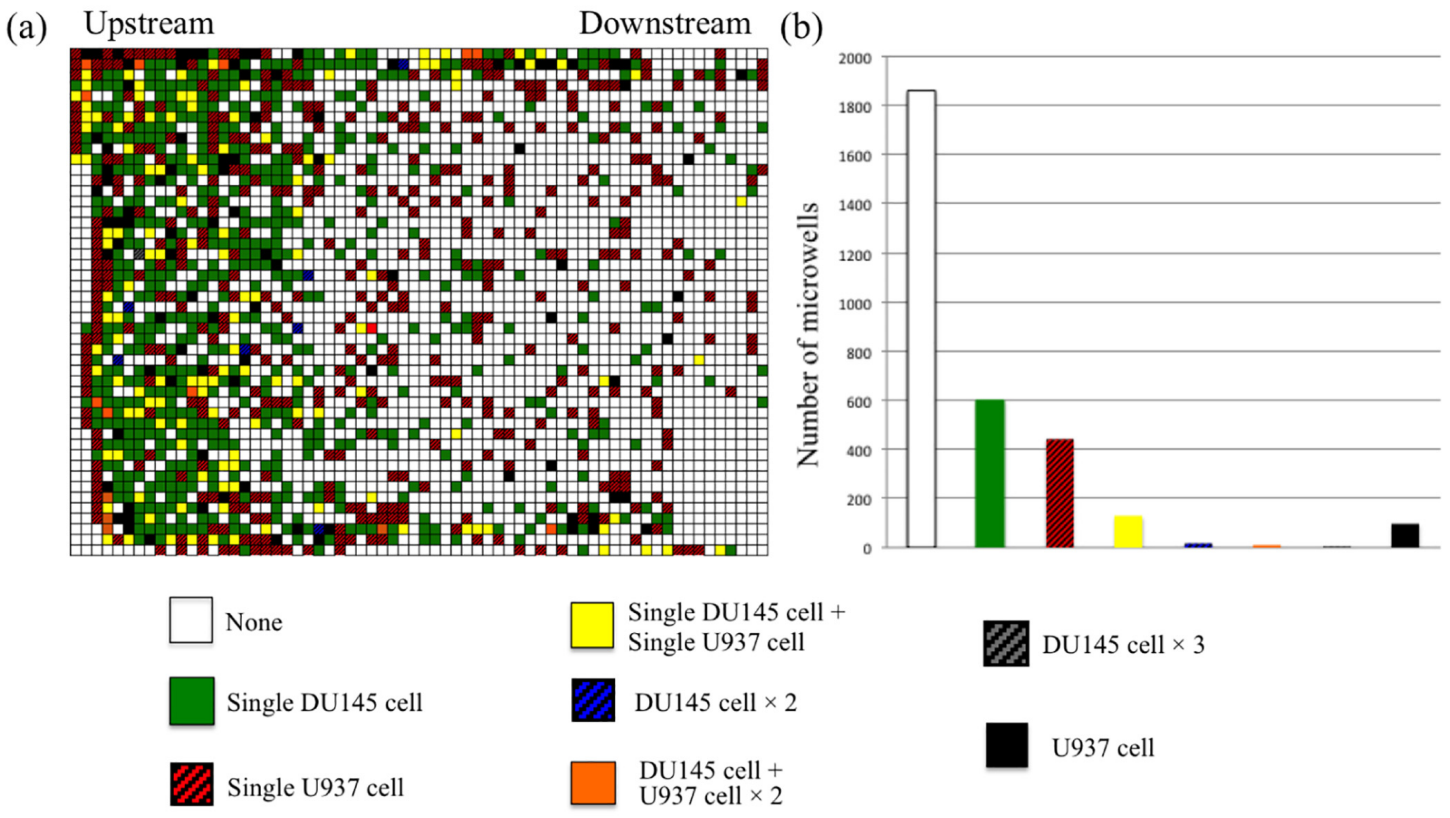
Result of trapping of DU145 cells and U937 cells on the device. (a) Pixel image of microwell on the device. Each pixel correspond each microwell on the device and (b) Distribution of contents of trapped cells in microwell. Colors show contents of trapped cells in microwell.

Trapping performance on the different cell lines was investigated by counting the number of trapped cells in microwells after immunostaning. Fig 5A shows the trapping pattern of each of the cell types, where the image represents the whole array in the device and each pixel represents each microwell. DU145 cells tend to be trapped at the upstream of the microwell array while U937 cells randomly distributed on the microwell array. The reason would be the larger diameter of DU145 cells than that of U937 cells, generating larger DEP force as shown in Eq (1). Since the force induced by DEP is proportional to the cubic of cell radius, DU145 cell receives larger force than U937 cell. The total number of trapped DU145 cells and U937 cells were 763 and 801, respectively. 39% of introduced cells were trapped into the microwells, and 33% of the microwells were occupied by single cell. Since the present electroactive microwells were designed to efficiently trap single DU145 cell of which diameter is bigger than that of U937 cell, the device shows good performance on single DU145 cell trapping, where 728-wells were occupied by single DU145 cells (598-wells were occupied by single DU145 cells, and 130-wells were occupied by single DU145 cells and single U937 cells). Only 15-wells were occupied by two or three DU145 cells. However, multiple U937 cells can be easily trapped into a same microwell since U937 cell (11 μm in diameter) is smaller than that of DU145 cell.

### Viability and Apoptosis Assay

**Fig 6:**
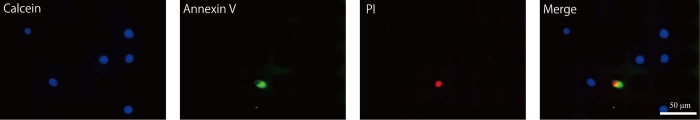
Viability and apoptosis assay. Annexin V (green) stains apoptotic cell, PI (red) stains dead cell and Calcein (blue) stains viable cells. Merged image identify both apoptotic and dead cell (green+red). Images of cells trapped in the microwells after 6 hours of incubation.

We investigated the viability of trapped cancer cells to detect and selectively analyze viable CTCs that could be involved in metastasis. Since almost all of the cancer cells in the blood are killed by cytotoxic cells such as T-cells, etc., and dead due to the anoikis or the damage caused by shear stress [22], it is highly required to identify viable cancer cells among a population of cells for further analysis. To check the viability of cancer cells, DU145 cells were trapped into the microwells and cultured in the device by introducing culture medium. After 6 hours, the trapped cells were stained with calcein (blue) for viable cells, annexin V (green) for apoptosis cells or PI (red) for dead cells. Almost all of the trapped cells (85%) emit blue fluorescence, indicating that the cells are viable even after 6 hours of incubation on the device (Fig 6).

### Fluorescent In Situ Hybridization (FISH)

**Fig 7:**
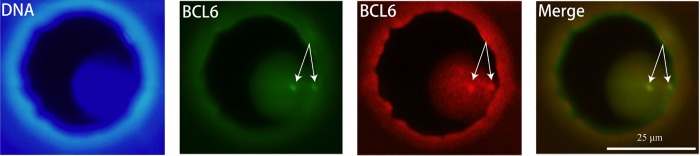
Fluorescence in situ hybridization analysis of BCL-6 gene in trapped PC3 cell. The arrows indicate signal of BCL6 probe. Green signal shows downstream part of BCL6 gene and red signal shows upstream part of BCL6 gene. Merge image shows yellow signal because of red and green signals are in the same location. This means chromosome 3q27 doesn’t cause translocation.

We carried out on-chip FISH in EMA to localize the presence or absence of specific DNA sequences on chromosomes since cancer cells often induce chromosomal rearrangement for the drug resistance. For demonstration, B-Cell Lymphoma6 (BCL6; gene for inhibition of apoptosis [23]) gene rearrangement was assessed for cultured PC3 cells using on-chip FISH. The FISH-negative shows close proximity of red spots (upstream part of BCL6 gene) and green spots (downstream part of BCL6 gene). Whereas FISH-positive shows dual-color break-apart probe. Fig 7 shows the result of the FISH assay for a PC3 cell in the microwell array. Translocation of apoptotic gene in PC3 cells can successfully be checked by on-chip FISH, and chromosome 3q27 of the cell did not cause translocation because red and green spots appear in the same location.

## Conclusions

In this study, we proposed a new method for single cancer cell analyses using the EMA device. The device enabled us to conduct efficient single cell trapping and three kinds of biochemical assays; immunostaining, viability/apoptosis assay and FISH at the single-cell level. Since the trapped single cells could be stably held inside microwells, we carried out the whole process for the assays by sequentially injecting reagents without complicated valve or tubing systems. Our simple EMA device combined with highly sensitive analytical assays promises high-throughput and parallelized analyses of rare cancer cells in a population of other cell types. One can also apply this technique to screening a drug candidate for tumor treatment by monitoring the response of target cells through on-chip assays.
